# A Flexible 8-in-1 Microsensor Embedded in Proton Battery Stack for Real-Time Microscopic Measurements

**DOI:** 10.3390/membranes13060573

**Published:** 2023-06-01

**Authors:** Chi-Yuan Lee, Chia-Hung Chen, Sheng-Ming Chuang, Chin-Yuan Yang, Jia-Yu Hsu

**Affiliations:** 1 Department of Mechanical Engineering, Yuan Ze Fuel Cell Center, Yuan Ze University, Taoyuan 32003, Taiwan; 2 HOMYTECH Global Co., Ltd., Taoyuan 33464, Taiwan

**Keywords:** proton battery stack, micro-electro-mechanical systems, flexible 8-in-1 microsensor

## Abstract

According to the latest literature, it is difficult to measure the multiple important physical parameters inside a proton battery stack accurately and simultaneously. The present bottleneck is external or single measurements, and the multiple important physical parameters (oxygen, clamping pressure, hydrogen, voltage, current, temperature, flow, and humidity) are interrelated, and have a significant impact on the performance, life, and safety of the proton battery stack. Therefore, this study used micro-electro-mechanical systems (MEMS) technology to develop a micro oxygen sensor and a micro clamping pressure sensor, which were integrated into the 6-in-1 microsensor developed by this research team. In order to improve the output and operability of microsensors, an incremental mask was redesigned to integrate the back end of the microsensor in combination with a flexible printed circuit. Consequently, a flexible 8-in-1 (oxygen, clamping pressure, hydrogen, voltage, current, temperature, flow, and humidity) microsensor was developed and embedded in a proton battery stack for real-time microscopic measurement. Multiple micro-electro-mechanical systems technologies were used many times in the process of developing the flexible 8-in-1 microsensor in this study, including physical vapor deposition (PVD), lithography, lift-off, and wet etching. The substrate was a 50 μm-thick polyimide (PI) film, characterized by good tensile strength, high temperature resistance, and chemical resistance. The microsensor electrode used Au as the main electrode and Ti as the adhesion layer.

## 1. Introduction

With an increasing demand for renewable energy, large-scale energy storage equipment is receiving unprecedented attention because it can integrate intermittent renewable energy into the grid. One of the key challenges lies in the rapid change and daily fluctuations of renewable energy, so energy storage equipment is required for a quick response to mitigate the rise or fall in energy [1].

Niroumand et al. [2] developed an in-situ diagnostic tool for proton exchange membrane fuel cell (PEMFC) stack gas leaks and checked for hydrogen leakage by applying pressure to the cathode or anode terminal. Therefore, leak prevention, spillover prevention, and the diagnosis of related quantities for cell stacks is a major challenge in the design and development of cell stacks. Andrews et al. [3] extended the concept of proton battery into a proton stream reactor; the reactor is applicable in large-scale renewable energy hydrogen production. The proton stream reactor is a scaled-up proton battery, where its solid carbon electrode is replaced with a flowing slurry electrode. Seifi et al. [4] used the Pistacia plant to prepare activated carbon, which was synthesized with CuO into a composite. It was found that besides the Brunauer, Emmett, and Teller (BET) area, the pore size closest to the size of hydrogen molecules was more likely to react with the hydrogen molecules and thus achieve more effective hydrogen adsorption. Han et al. [5] optimized the process of the oxygen sensor and reduced the number of plate electrodes to a quarter, and the cost and volume were reduced. Reshetenko [6] enhanced the performance of proton exchange membrane fuel cells by controlling the gas flow field and changing the quality of oxygen supply. Liebisch et al. [7] prepared a three-electrode micro oxygen sensor, and found that there was no chemical reaction other than oxidation reactions if the electrode was controlled. Furthermore, approximately zero analyte consumption could be achieved by the oxygen supply spilled from the electrode. Zhou et al. [8] prepared a super-hydrophobic oxygen sensor using a diatom cone. This sensor is more able to endure extreme environments than conventional sensors, and it is suitable for measuring dissolved oxygen. Movahedi et al. [9] studied the effect of gas diffusion layer deformation induced by closing pressure on the gas distribution in the proton exchange membrane fuel cell. They found that appropriate closing pressure could make the temperature distribution in the proton exchange membrane fuel cell more uniform. Ambardekar et al. [10] used the ion spray method to spray SnO_2_ and WO_3_ as the sensing layer on hydrogen sensors, and observed through an electron microscope that the sensor doped with both SnO_2_ and WO_3_ had a heterostructure. This heterostructure contributed to reducing the working temperature of the gas-sensing layer. Chen et al. [11] studied the voltage uniformity of proton exchange membrane fuel cell stacks and performed aging experiments on the cell stack with AC. They found that when the provided current density was doubled, the voltage decay rate of the cell stack increased with time. Liu et al. [12] studied a proton exchange membrane fuel cell stack composed of 30 single cells. They found that the voltage uniformity became worse as the cell stack temperature rose, and the voltage uniformity was improved when the cell stack was in low-temperature and low-current loading conditions. Shen et al. [13] studied the replacement of a water cooling system with an air cooling system in a conventional proton exchange membrane fuel cell stack. They found that the cell stack had the best stability quality at low air velocities, and the voltage difference of each single cell increased obviously under high current loads. Li et al. [14] designed a proton exchange membrane fuel cell flow control system, which had a fast response time, accurate stability, and interference resistance. The proton battery and proton exchange membrane fuel cell (PEMFC) have similar structures and thin-film materials. Excessively dry gases can cause dehydration inside the membrane electrode assembly (MEA), so water management is an extremely important control factor [15]. The gas diffusion layer, runner plate, cathode catalyst layer design, and battery operating conditions are the factors that may cause water accumulation [16,17,18]. Li et al. [19] indicated that when the proton exchange membrane water electrolyzer produces hydrogen, the higher the activity of water molecules in the air is, the faster the performance decays under a high applied potential.

Therefore, this study used the MEMS technology to develop a micro oxygen sensor and a micro clamping pressure sensor, which were integrated into the 6-in-1 microsensor developed by this R&D team. Subsequently, a flexible 8-in-1 (oxygen, clamping pressure, hydrogen, voltage, current, temperature, flow and humidity) microsensor was developed and embedded in the proton battery stack for real-time microscopic measurement.

## 2. Process of Flexible 8-in-1 Microsensor

**Figure 1:**
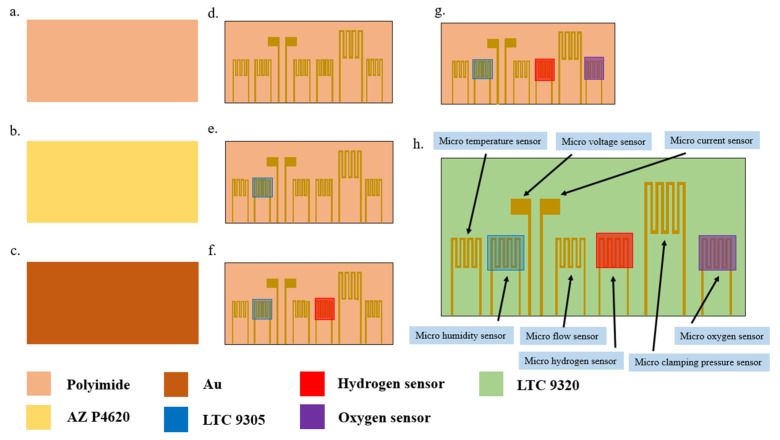
Fabrication process of flexible 8-in-1 microsensor.

**Figure 2:**
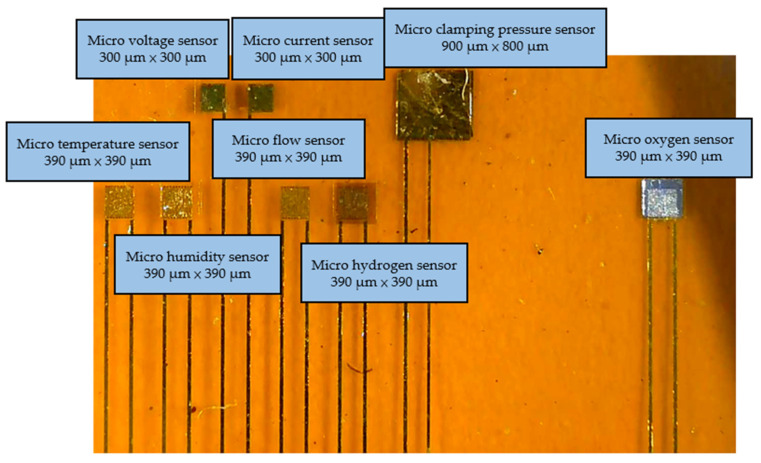
Optical micrograph of the flexible 8-in-1 microsensor.

Micro-electro-mechanical systems technologies were used in the process of developing the flexible 8-in-1 microsensor in this study, including physical vapor deposition (PVD), lithography, lift-off, and wet etching. The substrate was a 50 μm-thick polyimide (PI) film, characterized by good tensile strength, high temperature resistance, and chemical resistance. The microsensor electrode used Au as the main electrode and Ti as the adhesion layer. It is difficult to control the time parameter of microsensors fabricated by hand. However, while the wet etching process is relatively inexpensive, it is difficult to control the quality stability. At present, the wet etching process often shows over-etching due to the difficulty of time control. Over-etching can cause a large range of signal errors for each microsensor, leading to difficulties in integration with the back-end electrode and problems in achieving normalization. Therefore, wet etching was replaced by lift-off as the main electrode process in this study. The fabrication process of the flexible 8-in-1 microsensor is shown in Figure 1, and the optical micrograph is shown in Figure 2.

### 2.1. Flexible Printed Circuit Design for Flexible 8-in-1 Microsensor

**Figure 3:**
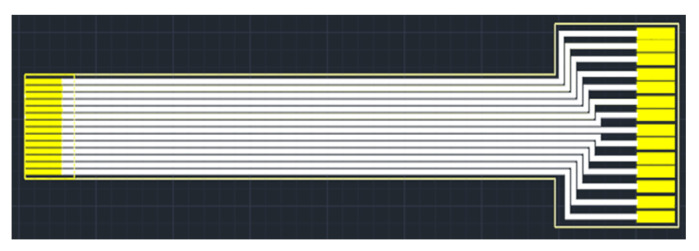
FPC design drawing.

**Figure 4:**
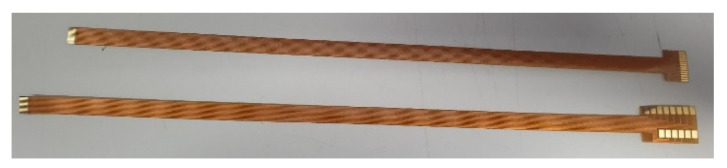
End product of FPC.

As the flexible 8-in-1 microsensor involves multiple different micro-electro-mechanical systems technology steps, the yield of microsensors is a major issue. Therefore, in order to reduce the cost of each flexible 8-in-1 microsensor, incremental short head sensors were used, and 112 microsensors could be produced at a time under the condition of 100% yield. To coordinate with this type of short head microsensor and reduce the difficulty of embedding it in the proton battery stack, this study followed the Flexible Printed Circuit (FPC) designed in the circle, drew the design drawing of the FPC using drawing software, and commissioned a manufacturer to produce it in batches. The design drawing and the end product of FPC are shown in Figure 3 and Figure 4, respectively.

### 2.2. Back-End Integration of Flexible 8-in-1 Microsensor

**Figure 5:**
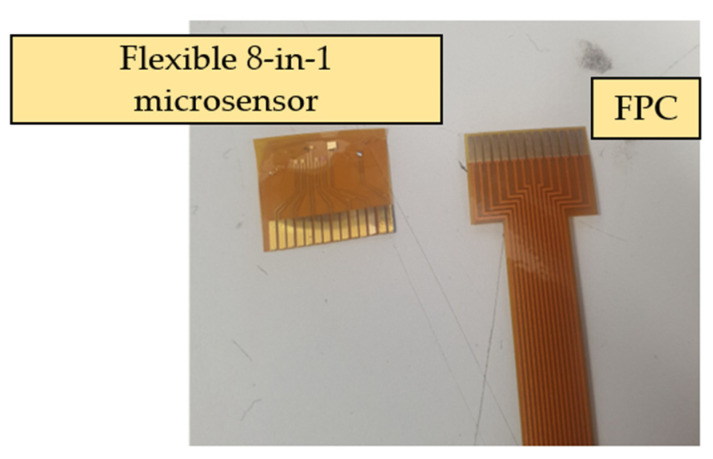
Completed flexible 8-in-1 microsensor and FPC.

**Figure 6:**
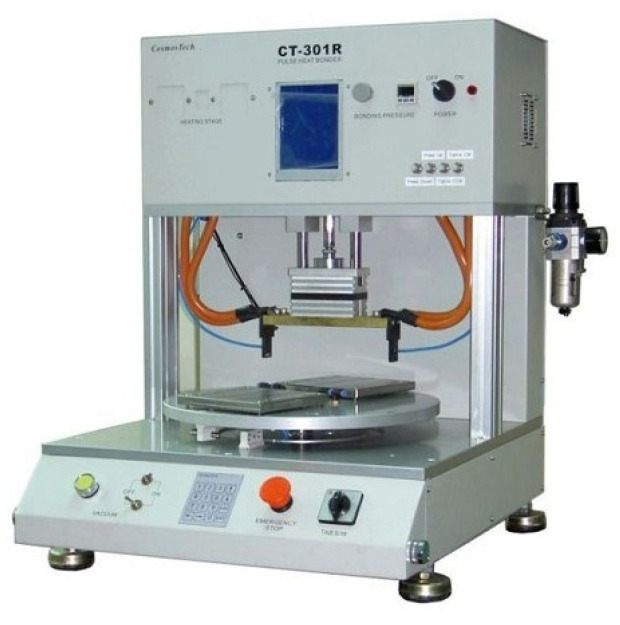
Pulsed electronic hot press.

**Figure 7:**
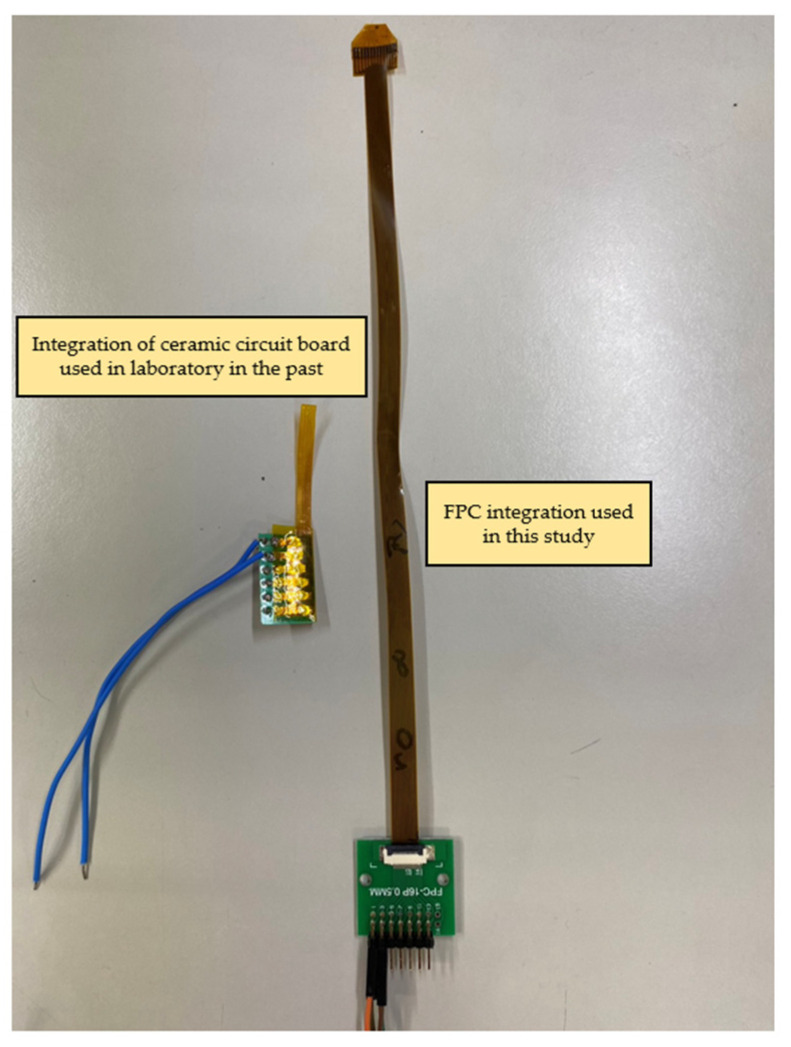
Comparison diagram of FPC back-end integration and conventional ceramic plate electrode integration in the laboratory.

The completed flexible 8-in-1 microsensor and FPC are shown in Figure 5. The two electrode assemblies were bonded using Anisotropic Conductive Film (ACF). The ACF is characterized by XY-axis insulation and Z-axis conduction. The region bonded using this method has both good strength and good conductivity. The hot press equipment employed was a pulsed electronic hot press. This machine can simultaneously provide the pressure and temperature required for conducting the anisotropic conductive adhesive, as shown in Figure 6. After the microsensor was bonded, the golden fingers were inserted into the flip-type electrode adapter plate to complete the back-end electrode integration of the microsensor. The back-end integration of the FPC used in this study and the conventional ceramic plate electrode integration in the laboratory are compared in Figure 7.

## 3. Correction of Flexible 8-in-1 Microsensor

**Figure 8:**
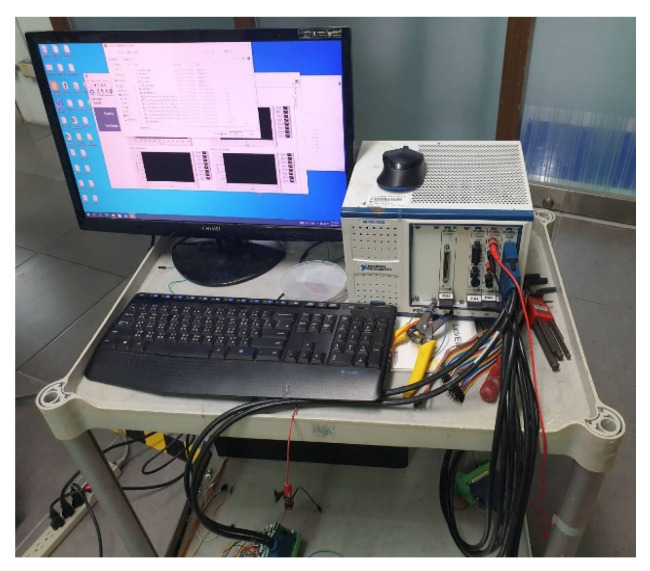
NI PXI 2575 digital acquisition unit.

**Figure 9:**
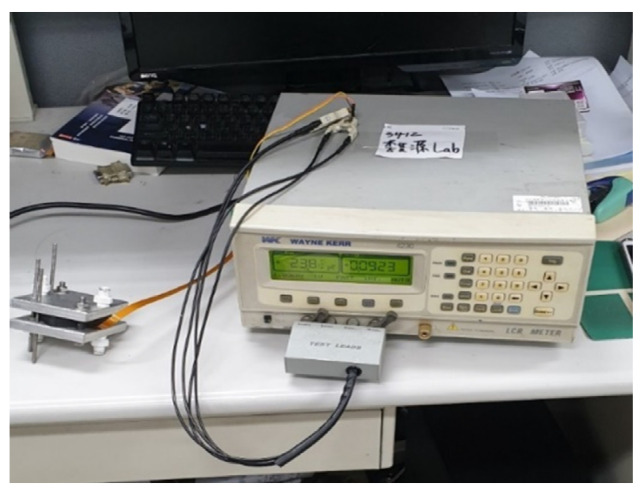
LCR meter.

In this study, the NI PXI 2575 (SHINING TECHNOLOGIES Co., Ltd., Hsinchu, Taiwan) digital acquisition unit of National Instruments (NI) (as shown in Figure 8) was used to capture the resistance and current variations of micro temperature, humidity, flow, hydrogen, and oxygen sensors. The data were extracted via the software control system designed by the LabVIEW system, and the correction curve was obtained from the extracted data for subsequent experiments. The variation in the micro clamping pressure sensor gave the capacitance, so the data were extracted using an LCR meter, as shown in Figure 9.

### 3.1. Temperature Correction of Flexible 8-in-1 Microsensor

**Figure 10:**
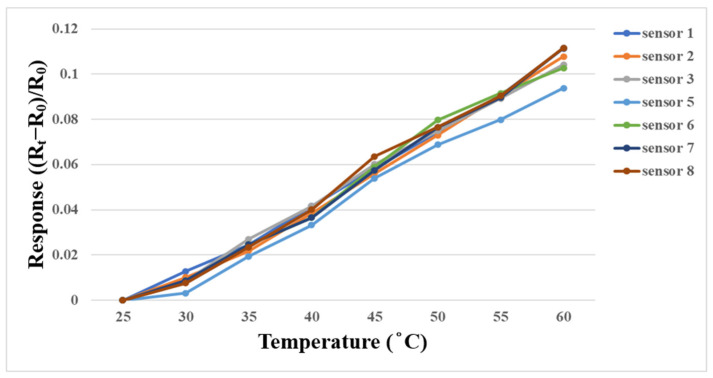
Correction curve of micro temperature sensor between 25 °C and 60 °C.

A DENG YNG^®^ DS45 (SCIENTIFIC INSTRUMENT Co., Ltd., New Taipei City, Taiwan) constant temperature oven was used for temperature correction in this study. The temperature as a variable for the performance test did not exceed 50 °C in subsequent experiments, so the temperature correction interval was 25 °C to 60 °C in this experiment, with the unit of °C. In order to simulate the actual experimental environment, the micro temperature sensor was embedded in the battery and then placed in the oven for correction. After the corrected temperature was stabilized, the resistance value in this interval was captured by the NI PXI 2575 data acquisition unit. Each micro temperature sensor was corrected three times and the average value was taken, as shown in Figure 10.

### 3.2. Humidity Correction of Flexible 8-in-1 Microsensor

**Figure 11:**
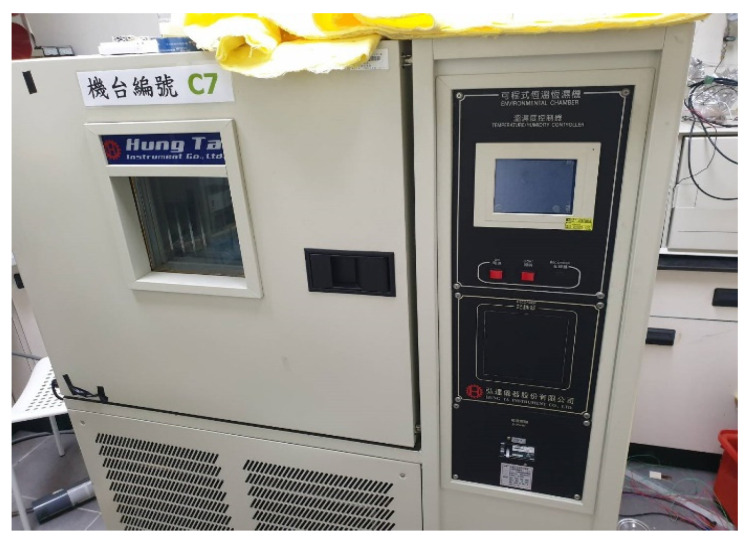
Program-controlled constant temperature and humidity testing machine Hung ta HT-8045A Environmental Chamber.

**Figure 12:**
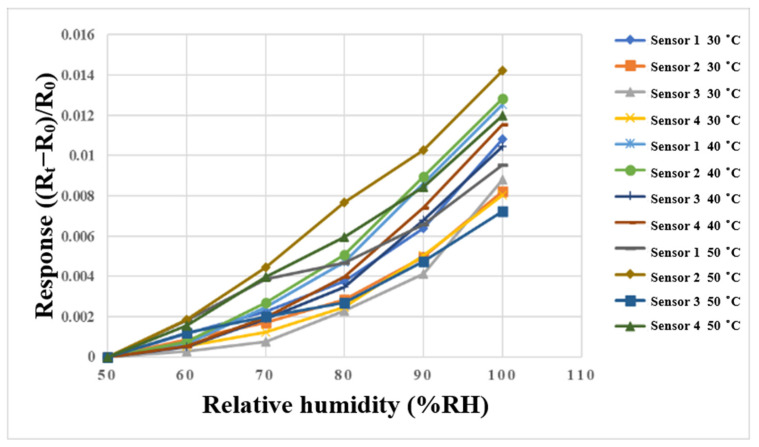
Correction curve of the micro humidity sensor between 50%RH and 100%RH.

A program-controlled constant temperature and humidity testing machine (Hung ta HT-8045A Environmental Chamber, Hung Ta Instrument Co., Ltd., Taichung, Taiwan, Figure 11) was used for humidity correction in this study. This experiment was performed using 30 °C, 40 °C, and 50 °C, the relative humidity was 50% to 100%, and the correction was performed at increments of 10%. In addition, in order to ensure the accuracy of the humidity displayed by the oven, two commercially available temperature and humidity sensors were used for correction experiments. The time interval of the NI PXI 2575 data acquisition unit was recorded when the values of constant temperature and humidity machine, and the two commercially available temperature and humidity sensors, were consistent. After 30 min of stabilization each time, the resistance value in the interval was captured by the NI PXI 2575 data acquisition unit. The correction curve is shown in Figure 12.

### 3.3. Flow Correction of Flexible 8-in-1 Microsensor

**Figure 13:**
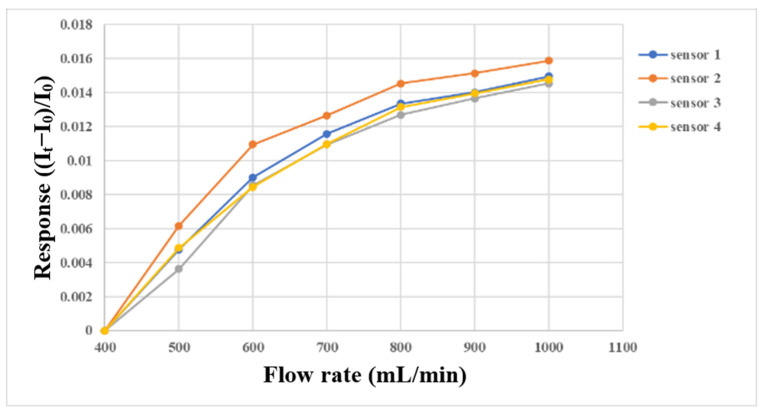
Micro flow sensor correction with gas supply between 400 mL/min and 1000 mL/min.

**Figure 14:**
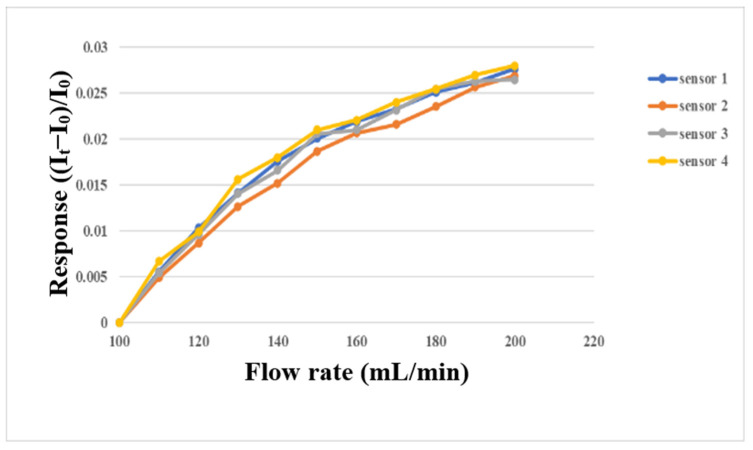
Micro flow sensor correction with water supply between 100 mL/min and 200 mL/min.

The micro flow sensor was embedded in the flow channel of the oxygen end runner plate with continuous fluid flow, because only this part had continuous fluid flow. First, the micro flow sensor was embedded in the battery to simulate the actual correction environment, and then the power supply, sensor, and NI PXI 2575 were connected in series. The power supply provided a stable 5 V voltage, the microsensor generated heat, and the current dropped. When the fluid flew, the generated heat was carried away to induce an increase in the current. At this time, the current variation was captured. The gas and fluid supply equipment in this experiment was an eight-channel machine, with the measurements conducted from 400 mL/min to 1000 mL/min at intervals of 100 mL/min. The gas flow correction results are shown in Figure 13. Water was used as the fluid for correction in this study, and the YOTEC WS (TOHAMA Co., Ltd., Hsinchu, Taiwan) Series variable speed flow pump was operated. The correction range was based on 100 mL/min, and the measurement was performed at intervals of 10 mL/min until 200 mL/min. The correction results are shown in Figure 14.

### 3.4. Hydrogen Correction of Flexible 8-in-1 Microsensor

**Figure 15:**
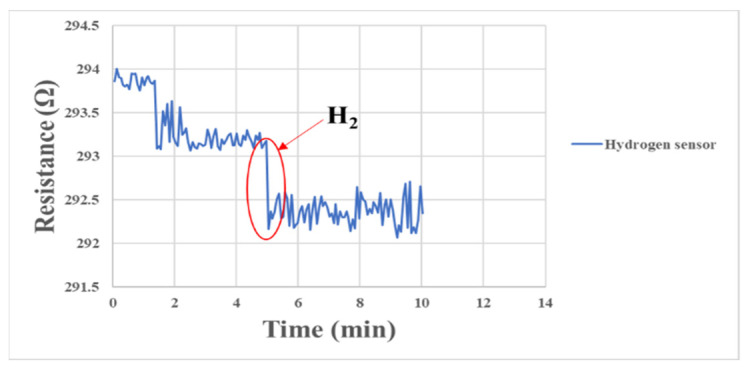
Resistance changes of micro hydrogen sensor correction between 0 min and 10 min.

The micro hydrogen sensor correction method and the micro oxygen sensor correction method in this study were similar to each other. The microsensor was embedded in the fuel cell for correction and sealed, and the gases were supplied by the eight-channel machine. When the surface of the micro hydrogen sensor was normal, the electrons were transferred from the test gas to the surface of the semiconductor film at the moment of contacting hydrogen, resulting in accumulative adsorption. When a large number of electrons accumulated on the surface of the semiconductor film, the conductivity of the semiconductor was increased, so the impedance of the microsensor decreased rapidly. The corresponding correction is shown in Figure 15.

### 3.5. Oxygen Correction of Flexible 8-in-1 Microsensor

**Figure 16:**
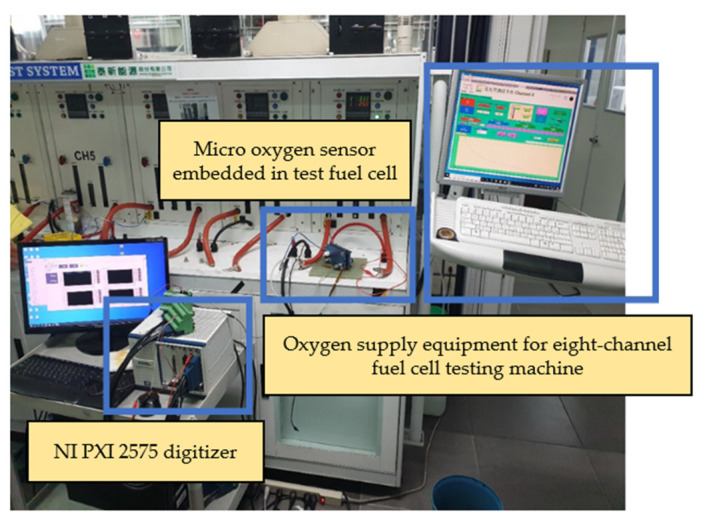
Experimental configuration of micro oxygen sensor correction.

**Figure 17:**
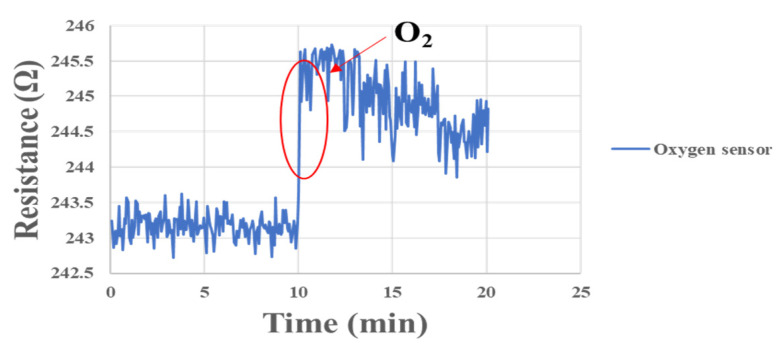
Resistance changes of micro oxygen sensor correction between 0 min and 20 min.

In order to simulate the oxygen flow in the flow channel, the micro oxygen sensor was embedded in the fuel cell flow channel for correction in this experiment. Furthermore, the oxygen provided by the eight-channel fuel cell testing machine was used for correction. The experimental configuration is shown in Figure 16. After the micro oxygen sensor was embedded in the battery, the oxygen at normal temperature and constant flow was admitted, and the resistance variation was captured by NI. When a high concentration of oxygen atoms (O) contacted the surface of a gas-sensing thin film, the electrons inside the material were captured to form adsorbates, e.g., oxygen ions (O-), leading to a depletion of the semiconductor surface. As a result, the electrons inside the gas-sensing thin film decreased, and the impedance of the sensing material increased rapidly, as shown in Figure 17.

### 3.6. Clamping Pressure Correction of Flexible 8-in-1

**Figure 18:**
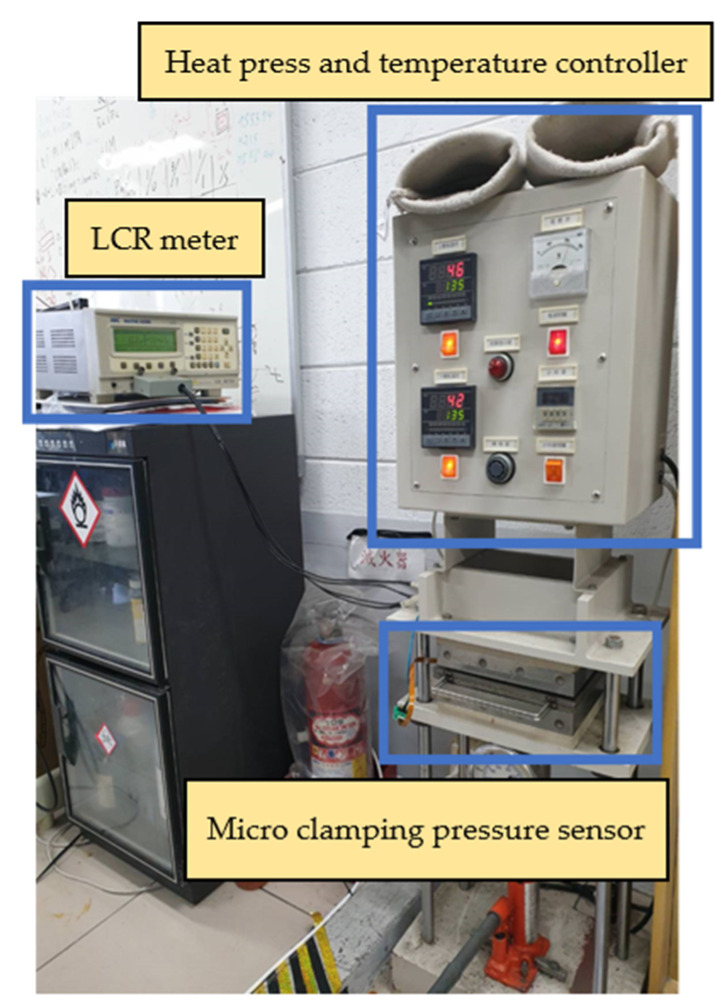
Experimental configuration of micro clamping pressure sensor correction.

**Figure 19:**
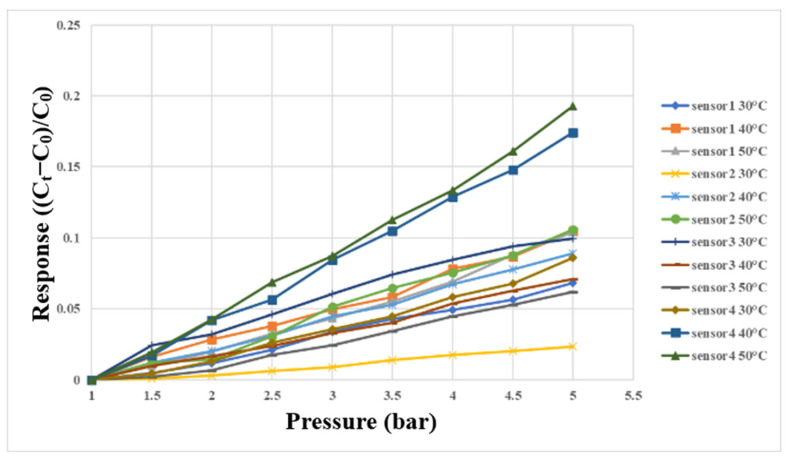
Correction curve of micro clamping pressure sensor between 1 bar and 5 bar.

The micro clamping pressure sensor in this study mainly measured the pressure of the runner plate ribs during the operation of the proton battery stack. First, the micro clamping pressure sensor was aligned with the ribs of the oxygen end runner plate and fixed. Afterwards, the LCR meter was connected to the back end of the microsensor, and the hot press provided the required pressure and temperature conditions for correction. The correcting instrument’s configuration is shown in Figure 18. The pressure range was from 1 to 5 bars at intervals of 0.5 bars, and the correction was performed at 30 °C, 40 °C, and 50 °C. The correction curve is shown in Figure 19.

### 3.7. Voltage and Current Correction of the Flexible 8-in-1 Microsensor

**Figure 20:**
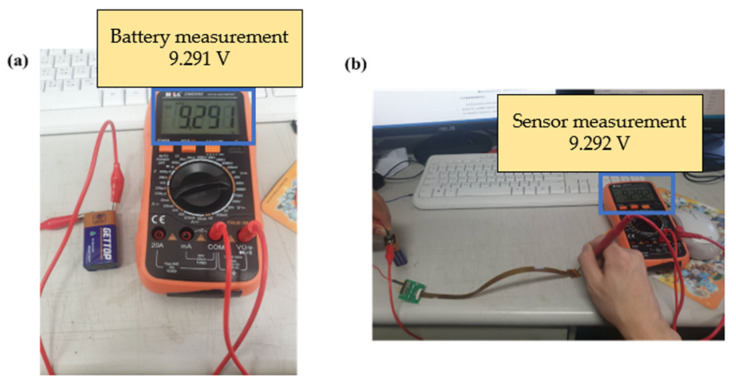
(**a**) Battery voltage measurement; (**b**) micro voltage sensor measurement battery.

**Figure 21:**
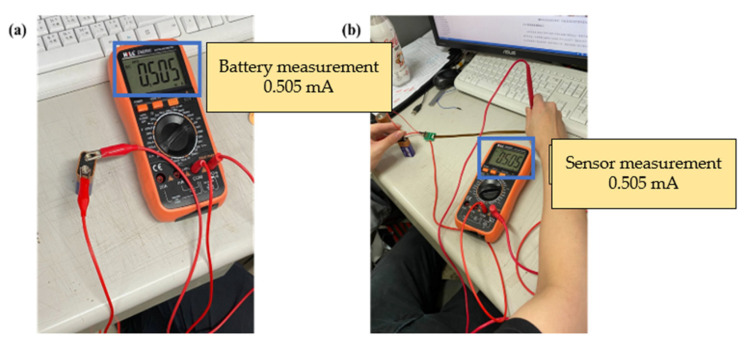
(**a**) Battery current measurement; (**b**) micro current sensor measurement battery.

This study used a digital multimeter to check whether the measurements of the micro voltage sensor and micro current sensor were correct. The battery voltage and current were verified by using the electric meter in the correction procedures, and then the micro sensing head was connected to the battery, as shown in Figure 20 and Figure 21. This connection ensured that the micro voltage sensor and micro current sensor could measure values correctly.

## 4. Flexible 8-in-1 Microsensor Embedded in Proton Battery Stack for Real-Time Measurement

**Figure 22:**
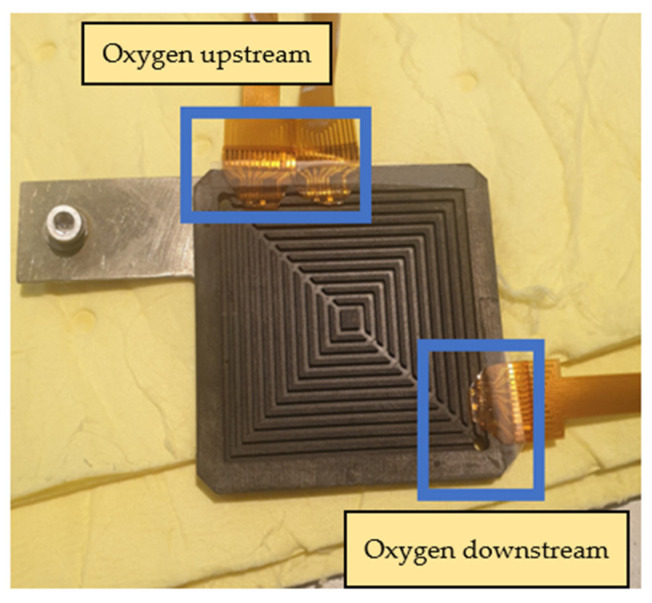
Position of flexible 8-in-1 microsensor embedded in oxygen end.

**Figure 23:**
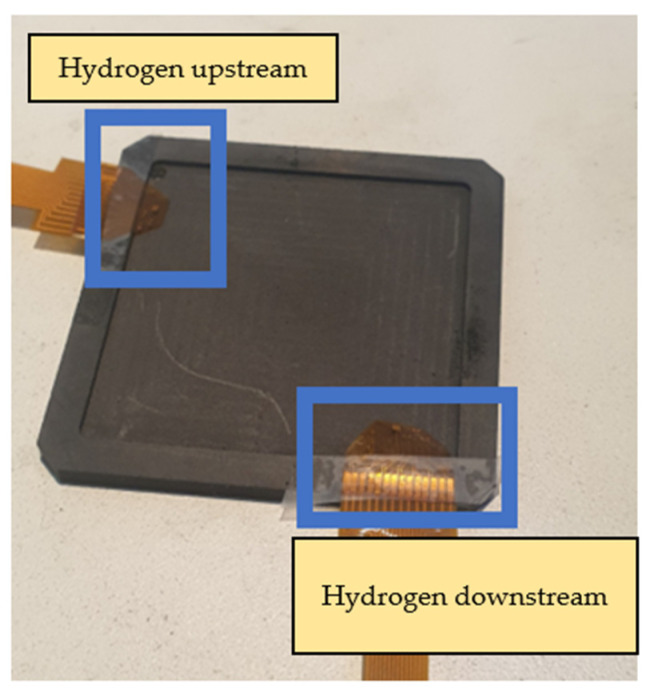
Position of flexible 8-in-1 microsensor embedded in hydrogen end.

The main body of the proton battery stack was jointly designed by the previous laboratory team and HOMYTECH Global Co., Ltd. [20]. The position where the flexible 8-in-1 microsensor is embedded is upstream and downstream of the oxygen terminals on both sides and upstream and downstream of the hydrogen terminals on both sides. A total of eight microsensors are embedded to measure and capture data at the same time, as shown in Figure 22 and Figure 23.

## 5. Conclusions

This study used MEMS technology to develop a flexible 8-in-1 microsensor on a 50 μm PI film substrate, which is characterized by a thin thickness, good softness, a small area, corrosion resistance, and chemical resistance. This study improved and enhanced the conventional mask of the multi-in-1 microsensor previously designed by this laboratory. The improvements include increasing the number of sensors of one set from 5 to 16, and replacing the wet etching with lift-off to define the electrode pattern. The enhancements greatly reduced the cost of each microsensor and greatly enhanced the stability. In terms of back-end integration, the ceramic plate electrode and copper wire back-end integration of the conventional mask were integrated and modified. Moreover, an FPC was designed and incorporated with DuPont wire and a slip cover adapter plate to move the weight of the microsensor to the back in order to reduce the possible difficulties that might arise in embedding the analyte. Furthermore, the volume of the microsensor was greatly reduced.

## Data Availability

Not applicable.

## Nomenclature

**Full Name**	**Abbreviation**
micro-electro-mechanical systems	MEMS
physical vapor deposition	PVD
polyimide	PI
proton exchange membrane fuel cell	PEMFC
membrane electrode assembly	MEA
Flexible Printed Circuit	FPC
Anisotropic Conductive Film	ACF
National Instruments	NI
